# Public Assemblies and Comsumption

**Published:** 1907-01

**Authors:** 


### Abstracts and Selections

### Public Assemblies and Comsumption.

Dispatches to the secular papers some days ago made much of some remarks of Dr. F. E. Daniel of Austin, Texas, on the peril of public assemblies in spreading disease germs, and naming especially those of tuberculosis. Doctor Daniel is President of the International Congress on Tuberculosis, and made his remarks at a meeting held in New York. He was specially severe on churches in this particular, using some strong figures of speech to describe them. If his words should be taken literally, they would create consternation in the public mind, and lead the timid to hesitate about going into public gatherings within doors. But it should be remembered that exaggeration is easy, and that specialists are likely to go to extremes. If the views expressed were literally correct and of very general application, we would be led to wonder that wholesale infection had not taken place to the serious decimation of the race. While the ravages of consumption and other contagious disease have been serious enough to awaken alarm and lead to increased care, no such results have followed as necessarily would be inferred from the statements referred to.

Nevertheless, this matter should not be passed over lightly. It has enough of truth in it to demand serious attention. Churches are not free from fault, in this matter, nor are public halls, schoolrooms, and other places where people gather in larger or smaller numbers. Ventilation is an art yet in its infancy, and a very sadly neglected infancy at that. Little has been said in a practical way about it, and less has been done. Janitors are far from being Solomons, and trustees are not all noted for carefulness of the duties of their office. Buildings are not constructed with any special reference to the supply of pure air for the lungs of those who occupy them.

In some cases there is the most criminal carelessness. People are crowded into rooms where it is impossible to give them proper ventilation without seriously inconveniencing some by drafts of cold air. As a result, they breathe the same air over and over again, until it becomes thoroughly vitiated and perilous. That there is danger in these things no one will deny. We name them now to urge upon all our people greater care in the ventilation of all our chiurches, as well as other assembly-rooms.—*Christian Advocate, Pittsburg, Pa.*

I should say that "wholesale infection" *has* "taken place, to the serious decimation of the race." Four hundred deaths a day—all the time. Referring to the last paragraph above: Keep it up *long enough* and it will be fatal, as was that "Black Hole of Calcutta."

